# Supplementary figures and images for: Single-cell RNA sequencing defines developmental progression and reproductive transitions of Pneumocystis carinii
Source: Microbiol Spectr. 2025 Aug 25;13(10):e01277-25. doi: 10.1128/spectrum.01277-25 (PMC12502742; doi:10.1128/spectrum.01277-25)

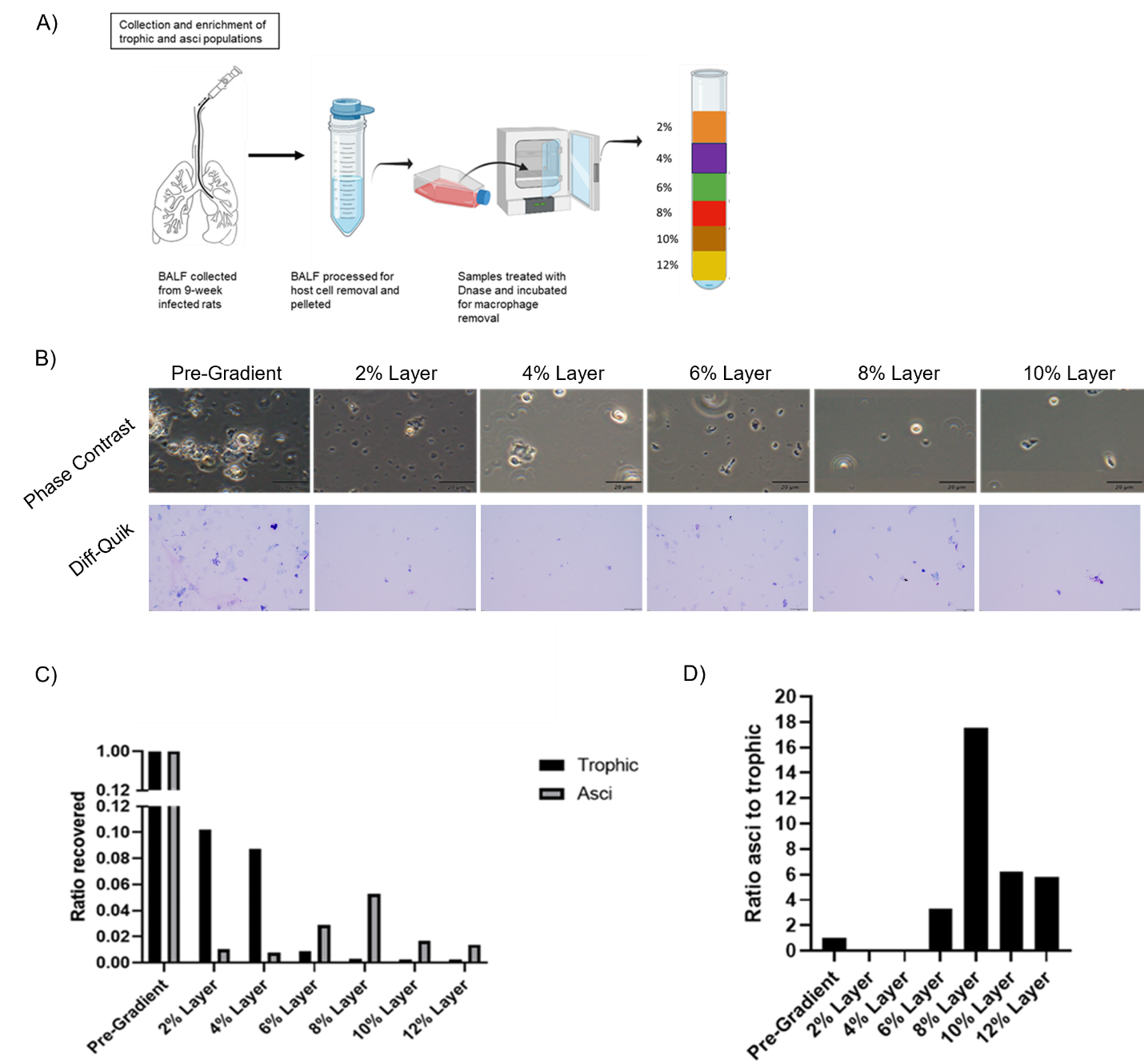

Supplement: Fig. S1 — Optimization of separation, enrichment, and viability for single-cell RNA sequencing of Pneumocystis carinii. [file spectrum.01277-25-s0001.tif]

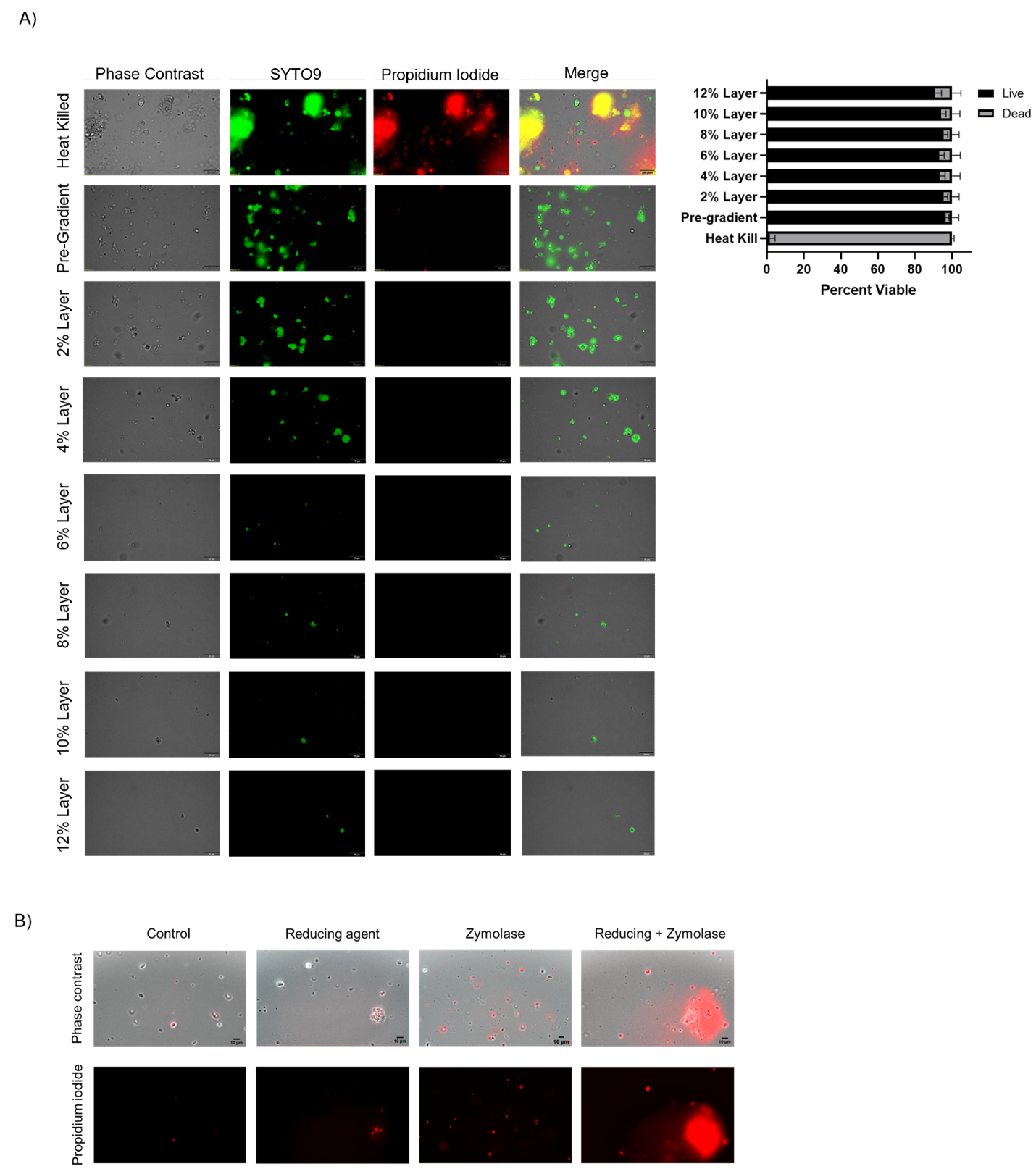

Supplement: Fig. S2 — Viability and lysis data. [file spectrum.01277-25-s0002.tif]
