# Supplementary material for: Single-cell RNA sequencing defines developmental progression and reproductive transitions of Pneumocystis carinii
Source: Microbiol Spectr. 2025 Aug 25;13(10):e01277-25. doi: 10.1128/spectrum.01277-25 (PMC12502742; doi:10.1128/spectrum.01277-25)
Supplement: Supplemental material — Supplemental methods; Tables S1 to S4. [file spectrum.01277-25-s0003.docx]

**Supplemental Methods**

**Optimization of Separation, Viability, and 10X Genomics Assay for Single-Cell RNA Sequencing of *Pneumocystis carinii***

To establish a robust protocol for single-cell RNA sequencing (scRNA-seq) of *P. carinii*, we optimized organismal enrichment, viability preservation, and compatibility with the 10X Genomics Chromium platform. Bronchoalveolar lavage fluid (BALF) from infected rats was placed into a Ficoll density gradient to reduce host cell background and enrich for developmental stages. Trophic nuclei were predominantly recovered from the 2% and 4% layers, while asci were enriched in higher-density layers (6–12%) (Supplemental Fig. S1B). Post gradient the individual layers were separated with a 3cc syringe and 16 gage metal canula and aspirated gently three times to break up cell clumping before being placed in media for viability assay.

All fractions maintained greater than 80% viability, as confirmed by dual-fluorescence staining immediately post-isolation and after a 2-hour incubation at room temperature to account for potential delays in transport or processing at core (Supplemental Fig. S1C). However, standard 10X lysis reagents were unable to efficiently lyse the ascus wall. Propidium iodide staining confirmed incomplete nuclear release in these stages unless zymolyase was added (Supplemental Fig. S1D), necessitating enzymatic digestion to ensure comprehensive representation across the life cycle.

Two pilot scRNA-seq runs informed the design of the final scRNA-seq run. In Pilot 1, we targeted 4,000 cells at a sequencing depth of 25,000 reads per cell, following 10X Genomics recommendations for non-Mammalian eukaryotes. However, overloading yielded 12,813 cells, compromising the sequencing depth (Supplementary Table ST1). To avoid oversampling and improve transcript detection sensitivity, particularly important for fungal species with compact transcriptomes and low mRNA content, we increased the target depth to 80,000 reads per cell in Pilot 2. Although 4,767 cells were recovered, the deeper coverage resulted in >25,000 usable reads per cell, which allowed sufficient resolution of developmental heterogeneity and reliable clustering (Supplementary Table ST2).

Based on these optimizations, our final experiment targeted 8,000 cells at 80,000 reads per cell, striking a balance between sequencing saturation and cellular diversity to ensure robust downstream analyses (Supplementary Table ST3).

**Enumeration of *P. carinii* Trophic and Ascus Forms**

*P. carinii* life cycle stages were quantified using established microscopy-based enumeration protocols. For each sample, three 10-µL aliquots were placed on microscope slides (Fisher Scientific, Pittsburgh, PA), air-dried, and heat-fixed by briefly passing the slides through an open flame three times, each for ~1 second. To distinguish developmental forms, asci were visualized using cresyl violet acetate (CVA; Sigma-Aldrich, St. Louis, MO), while total nuclei, including trophic and ascus forms, were stained using a modified Wright-Giemsa stain (Leuko-stat; Fisher Scientific, Pittsburgh, PA). Counts from all three fields were averaged to determine the concentration and ratio of developmental stages (1)The final cell suspensions were normalized to a target concentration of 700 cells/µL for loading into the 10X Genomics single-cell RNA-seq assay per the manufacturer's suggestion and core consultation.

**Microscopic Evaluation and Viability Assessment of *P. carinii***

The viability of *P. carinii* was assessed using individual nucleic acid stains, SYTO 9 and propidium iodide (Thermo Fisher Scientific, Waltham, MA), following the manufacturer’s protocols for dual fluorescent labeling (2). Samples were incubated with the staining solution for 15 minutes at room temperature in the dark. A heat-killed control, incubated at 80°C for 30 minutes, was included to validate staining specificity and to distinguish viable from nonviable cells. Three replicate slides were prepared for each density gradient layer, and a minimum of 200 cells per sample were evaluated by fluorescence microscopy. This method provided a robust and reproducible viability assessment across enriched fungal populations.


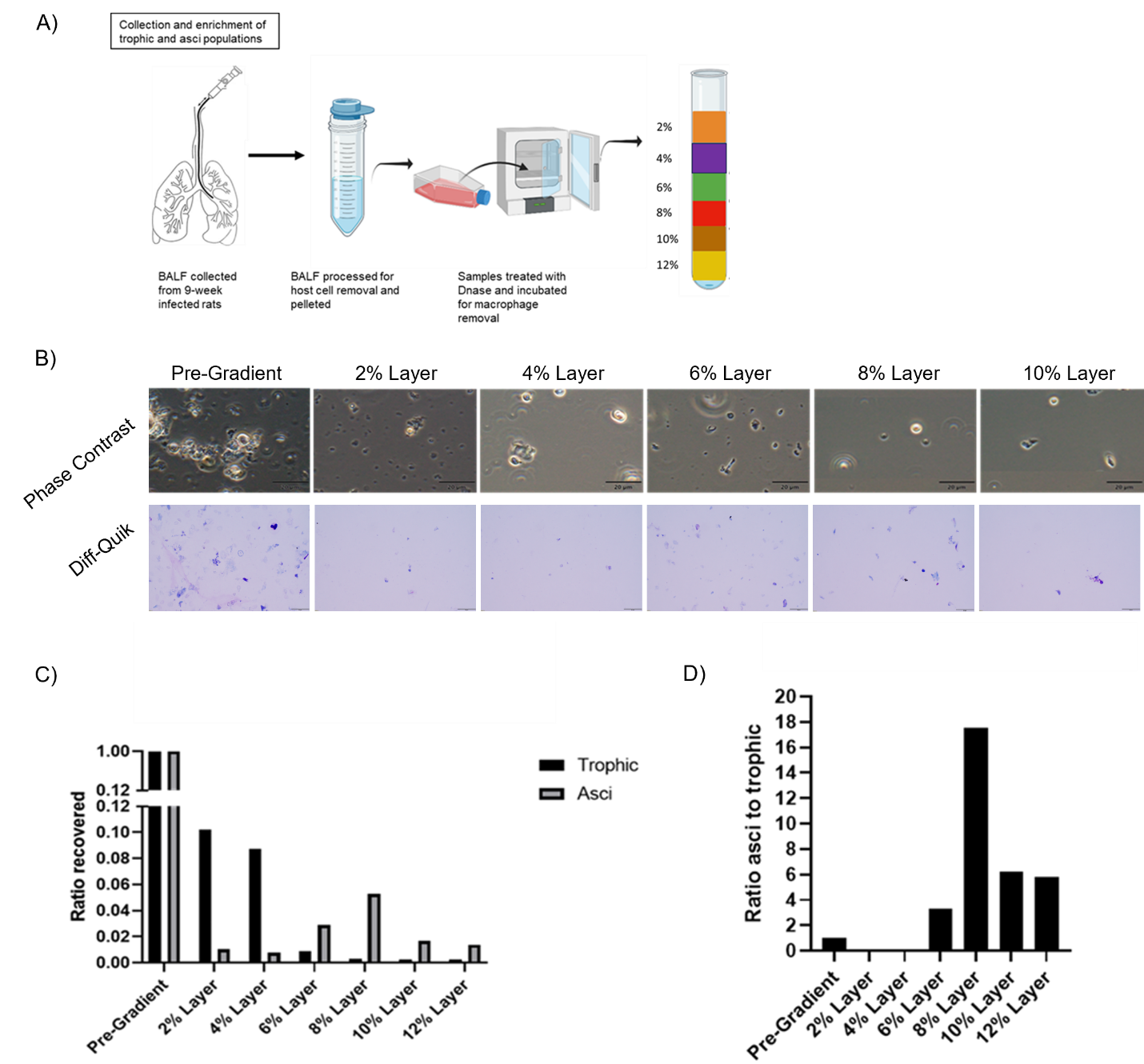


**Supplemental Figure 1.** Optimization of separation, enrichment, and viability for single-cell RNA sequencing of *Pneumocystis carinii*. **Panel A:** Schematic overview of the single-cell preparation pipeline. Bronchoalveolar lavage (BAL) fluid was collected from heavily infected rats and processed via differential centrifugation followed by Ficoll-Hypaque gradient separation (2–12%). **Panel B:** Representative phase-contrast images showing preservation of both trophic and ascus forms across Ficoll layers, suitable for downstream transcriptomic analysis (scale bars, 20 μm). Wright-Giemsa staining is shown with green arrows indicating trophic forms and red arrows indicating asci containing developing spores. Insets show higher magnification of maturing asci (scale bars, 10 μm). **Panel C:** Proportional composition of each fraction, showing the relative percentage of trophic forms (black) and asci (gray) within each layer. Asci accounted for 75–95% of the population in the 6–12% fractions, indicating effective separation of late-stage forms. **Panel D:** Ascus-to-trophic cell ratio is calculated for each layer. Ratios
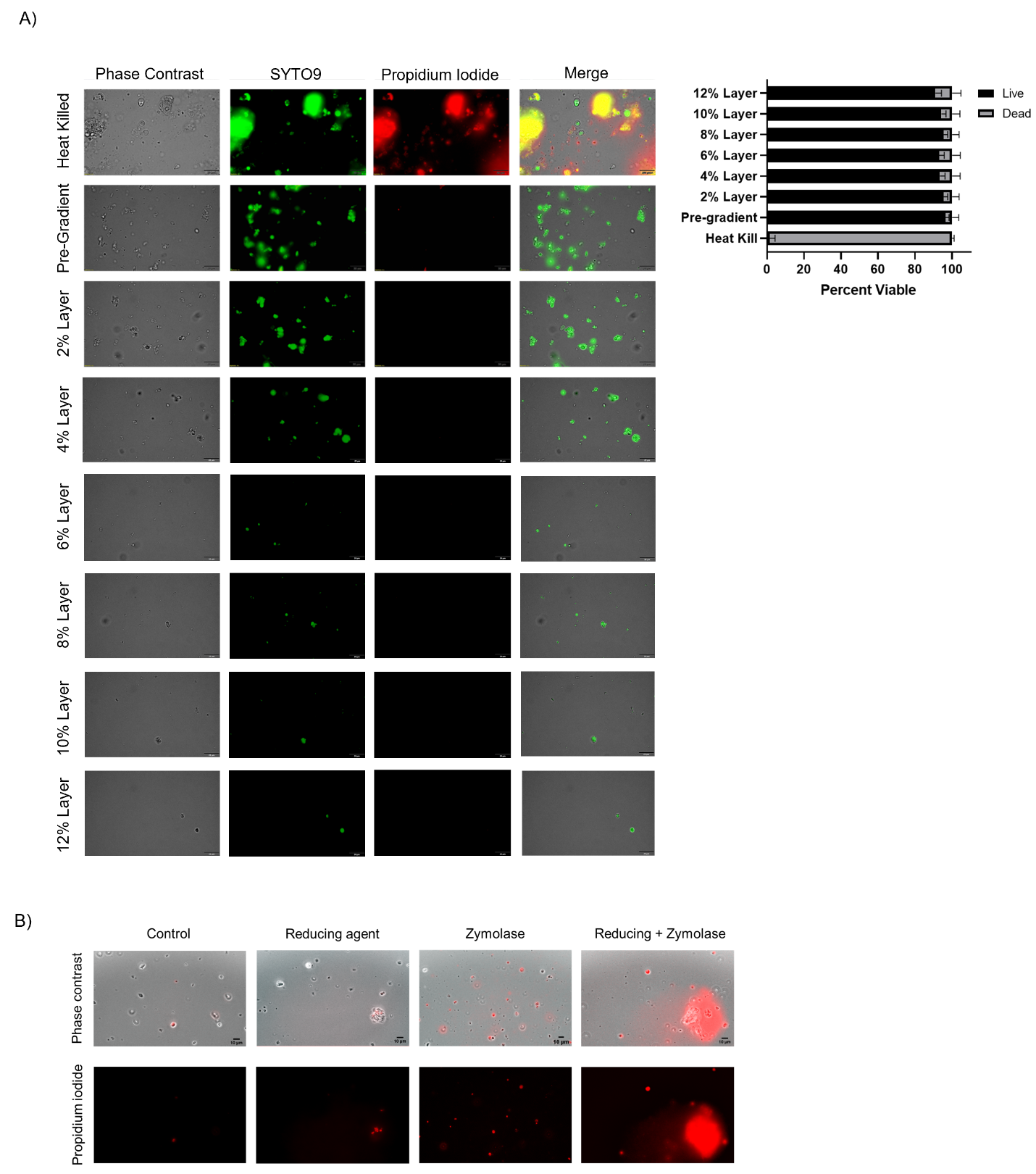
ranged from ~3:1 in the 6% layer to greater than 17:1 in the 12% layer, confirming the strong enrichment of mature asci in higher-density fractions. Data represent the mean of biological replicates from BALF of heavily infected rats.

**Supplemental Figure 2.** Assessment of Cell Viability and Ascus Lysis Efficiency in *Pneumocystis carinii* Preparations. **Panel A:** Viability data assessed using SYTO 9 and propidium iodide staining. Green fluorescence indicates live cells with intact membranes, while red fluorescence marks dead or lysed cells. All gradient layers maintained a viability of greater than 80% after separation and following two hours of incubation at room temperature. **Panel B:** Evaluation of lysis efficiency under four treatment conditions: untreated, reducing agent only, zymolyase only, and combined reducing agent plus zymolyase. Propidium iodide fluorescence (bottom row) demonstrates optimal nuclear release under the combined condition, while corresponding phase-contrast images (top row) show structural disruption of thick-walled asci (scale bars, 10 μm).

Supplementary Table ST1 Pilot 1: Sequencing data

| Sample | Cells recovered | Reads per Cell |
| --- | --- | --- |
| 2% Layer Sample | 6,388 | 5,396 |
| 6% Layer Sample | 2,393 | 14,045 |
| 8% Layer Sample | 2,706 | 11,383 |
| 10% Layer Sample | 1,326 | 25,529 |

Supplementary Table ST2 Pilot 2: Sequencing data

| Sample | Cells recovered | Reads per Cell |
| --- | --- | --- |
| 2%- & 4%-Layer Sample | 2,582 | 34,650 |
| 6%- & 8%-Layer Sample | 1,258 | 91,153 |
| 10%- & 12%-Layer Sample | 927 | 116,339 |

Supplementary Table ST3 *P. carinii* infection Transcriptome: Sequencing data

| Sample | Cells recovered | Reads per Cell |
| --- | --- | --- |
| 2%- & 4%-Layer Sample | 1,412 | 808,500 |
| 6% Layer Sample | 16,121 | 63,585 |
| 8% Layer Sample | 19,732 | 58,032 |
| 10%- & 12%-Layer Sample | 3,900 | 323,452 |
| 2%- & 4%-Layer Sample | 12,636 | 101,600 |
| 10%- & 12%-Layer Sample | 33,915 | 33,638 |

Supplementary Table ST4: RT-qPCR Primers

| **Name** | **Sequence 1** | **Sequence 2** |
| --- | --- | --- |
| T552_01932 | CAACAATGCCTCCTCGACCT | GCATTCACCAGCGCATCATT |
| T552_01043 (*dmc1*) | AATGGGAGGGGCAGAAGGTA | GAGCTCTCCTCGTCCAGAGT |
| T552_01968 (*mcp2*) | TGGGGGTTTTGCCTCCAAAT | ATGCTTCCAATGCTGCTCCT |
| TS | CATGGGGAAGACCGCCCTGA | CTCCACGTATAAAAACTCGCTTGGT |
| T552_04188 | CTGTACTTGCTAAAACTGGGTGTGT | ACCGCGACACGTCGAACTCT |
| T552_00947 | AGGCAACGGGCAGGACGTCAA | TCCTGACCGTGATCCTGATGGAAGC |

**References**

1. Ruffolo JJ, Cushion MT, Walzer PD. 1986. Techniques for examining *Pneumocystis carinii* in fresh specimens. J Clin Microbiol 23:17-21.

2. Deng Y, Wang L, Chen Y, Long Y. 2020. Optimization of staining with SYTO 9/propidium iodide: interplay, kinetics and impact on *Brevibacillus brevis*. Biotechniques 69:88-98.
